# Submerged Macrophytes Mitigate Direct and Indirect Insecticide Effects in Freshwater Communities

**DOI:** 10.1371/journal.pone.0126677

**Published:** 2015-05-15

**Authors:** William R. Brogan, Rick A. Relyea

**Affiliations:** 1 Smithsonian Environmental Research Center, Edgewater, Maryland, 21037, United States of America; 2 Rensselaer Polytechnic Institute, Troy, New York, 12180, United States of America; Gettysburg College, UNITED STATES

## Abstract

Understanding how ecological interactions mitigate the impacts of perturbations such as pesticides in biological communities is an important basic and applied question for ecologists. In aquatic ecosystems, new evidence from microcosm experiments suggests that submerged macrophytes can buffer cladocerans from pulse exposures to the widely used insecticide malathion, and that mitigation increases with macrophyte density. However, whether these results scale up to more complex aquatic communities where ecological interactions such as competition can alter toxicity is unknown. Further, macrophyte abilities to mitigate different insecticide exposure scenarios (i.e. single versus repeated pulses) have never been tested. To address these gaps, we performed a factorial mesocosm experiment examining the influence of four macrophyte treatments (0, 10, 50, or 100 *Elodea Canadensis* shoots planted per mesocosm) crossed with three malathion exposure scenarios (no insecticide, single pulse, repeated pulses) on aquatic communities containing zooplankton, phytoplankton, periphyton, two snail species, and larval amphibians. In the absence of macrophytes, single malathion pulses caused short-term declines in cladoceran abundance followed by their rapid recovery, which precluded any indirect effects (i.e. trophic cascades). However, repeated malathion pulses caused cladoceran extinctions, resulting in persistent phytoplankton blooms and reduced abundance of one snail species. In contrast, with macrophytes present, even at low density, malathion had no effect on any taxa. We also discovered novel effects of macrophytes on the benthic food web. In the two highest macrophyte treatments, we observed trends of reduced periphyton biomass, decreased abundance of one snail species, and decreased amphibian time to and mass at metamorphosis. To our knowledge, this is the first evidence of negative submerged macrophyte effects on amphibians, a taxa of global conservation concern. Our findings suggest that facilitating macrophytes could be an important strategy for buffering freshwater communities from insecticides, though consideration of their impacts on animal species is necessary.

## Introduction

A contemporary challenge facing ecologists and ecotoxicologists is to elucidate factors that can mitigate the effects of anthropogenic contaminants in aquatic ecosystems. Traditionally, the ecological effects of contaminants, such as insecticides, are based on laboratory toxicity studies using a small number of test species and then extrapolated to entire communities [1]. However, such tests are designed to eliminate sources of environmental variation e.g., [2,3], but accumulating evidence suggests this can lead to discrepancies between predicted and actual insecticide effects in nature [4–6].

One common cause of discrepancy between predicted and actual effects of insecticides is the influence of ecological interactions. For example, in freshwater communities, natural stressors such as competition and predation can exacerbate direct insecticide toxicity [7–10]. Further, at concentrations that traditional toxicity tests predict should have no effect, insecticides can affect the growth and survival of relatively resistant species through indirect trophic interactions. For example, low concentrations of many insecticides decimate cladoceran zooplankton, initiating trophic cascades that cause phytoplankton blooms. As the phytoplankton bloom, they shade the benthos, reduce periphyton growth, and adversely affect benthic grazer growth and survival [11–13]. While a preponderance of studies in aquatic ecosystems have targeted factors that exacerbate insecticide effects, comparatively few studies have identified factors that might mitigate these effects.

Growing evidence suggests that submerged macrophytes can mitigate insecticide effects in aquatic ecosystems. For example, Brogan & Relyea [14] demonstrated that realistic densities of a cosmopolitan submerged macrophyte, Canadian waterweed (*Elodea canadensis*), caused up to nine-fold reductions in the toxicity of the insecticide malathion to the cladoceran *Daphnia magna* in small (0.95-L) outdoor jars. Moreover, each increase in macrophyte density caused greater mitigation. We also understand the mechanism of this mitigation. As submerged macrophytes photosynthesize, they increase water pH by taking up CO_2_, which decreases carbonic acid concentration and shifts the bicarbonate buffer system towards the more alkaline bicarbonate and carbonate [15]. This higher pH causes malathion to degrade more rapidly via alkaline hydrolysis [14,16]. However, as the mitigating effects of submerged macrophytes on insecticide toxicity have thus far only been documented at the microcosm scale, a critical next step is to examine whether these effects occur in more spatially and ecologically complex communities.

In addition to mitigating insecticide direct effects, submerged macrophytes may also dampen the indirect effects of insecticides in freshwater communities. For example, macrophytes can suppress phytoplankton growth via allelopathy [17] and aqueous nutrient competition [18,19]. Thus, even if zooplankton decline following insecticide exposure, submerged plants may still prevent phytoplankton blooms. While predicting the impacts of macrophytes on phytoplankton is relatively straightforward, their effects on periphyton and grazers (e.g., snails and larval amphibians) are still poorly understood. For example, while increasing habitat complexity created by macrophytes is known to reduce fish and macroinvertebrate predation on snails and tadpoles [20–24], no studies to our knowledge have examined the impact of submerged macrophytes on the abundance and growth of snails and larval amphibians in the absence of predators. Thus, there is a need for studies examining the influence that macrophytes have on grazers, particularly in the presence of perturbations like insecticides.

When examining the influence of macrophytes on insecticide effects in aquatic communities, there is also a need to consider different insecticide-exposure scenarios. For example, depending on weather patterns and application frequencies, insecticide exposure in aquatic communities can occur as single or repeated pulse perturbation events [25–27]. However, studies examining community responses to different insecticide exposure scenarios have received little attention. In the few studies investigating the ecological effects of single and repeated insecticide perturbations, repeated- exposures have ecological effects that are longer lasting and many times larger in magnitude than single exposures [12,28,29]. While submerged macrophytes can mitigate single insecticide applications in microcosm studies [30,31], their ability to mitigate the effects of repeated exposures has never been examined. Clearly, considering different exposure regimes is critical for understanding the factors influencing realistic insecticide impacts in freshwater communities.

To address these gaps in our understanding, we examined the mitigating role of a range of natural macrophyte densities in freshwater communities containing phytoplankton, periphyton and 22 species of animals (zooplankton, snails, and larval amphibians) during several realistic insecticide-exposure scenarios. We used the organophosphate insecticide malathion (Diethyl 2-dimethoxyphosphorothioylsulfanylbutanedioate) because it is one of the most commonly used active ingredients in the U.S. [32], with 10–14 million kg applied annually (National Pesticide Use Database [33], www.ncfap.org/database/national.php). Despite malathion’s popularity, few studies have examined its ecological effects in non-target communities. We hypothesized that the magnitude of malathion’s direct and indirect effects would increase with the number of insecticide exposure events (control < single pulse < repeated pulses) and that these effects would decrease with increasing submerged macrophyte density.

## Materials and Methods

### Experimental design

The experiment was conducted in Summer 2009 at University of Pittsburgh’s Pymatuning Laboratory of Ecology. We used a completely randomized, factorial design crossing four macrophyte treatments (0, 10, 50, and 100 *E*. *canadensis* shoots initially planted per mesocosm) with three malathion exposure scenarios (no insecticide, a single application, and repeated applications every three wks). The 12 treatment combinations were replicated four times for a total of 48 experimental units. This study was approved by the University of Pittsburgh's Institutional Animal Care and Use Committee (IACUC) under Protocol #09–04536. None of the species used in this study are protected or endangered.

### Experimental setup

The experimental units were outdoor 1,200-L mesocosms containing 850 L of well water. On 2 May, we added 95 L of sediment to each mesocosm and on 28 to 31 May, we haphazardly planted the appropriate number of *E*. *canadensis* (*hereafter Elodea*) shoots throughout each mesocosm, attempting to evenly spacing the shoots. Though we haphazardly selected *Elodea* shoots for planting, we attempted to stock each mesocosm with shoots spanning a similar range of masses. We simulated planting in the 0-macrophyte treatment to standardize sediment disturbance. The *Elodea* shoots used in this experiment were collected and mixed from three local state-owned wetlands with approval and permits from the Pennsylvania Fish and Boat Commission. Prior to adding the *Elodea* shoots to the mesocosms, we placed the collected plants in 200-L wading pools containing well water and sediment for 2 wks to allow any attached invertebrate eggs to hatch.

On 21 May, we established microbial, algal and zooplankton communities in each mesocosm. To do this, we directly collected water using buckets from each same pond where we had collected *Elodea*. We also used a zooplankton tow (250 μm mesh) to collect zooplankton. We combined all water and zooplankton samples, removed all predatory invertebrates, and then added 200-mL aliquots of this water to each mesocosm. On 26 May, we added five unglazed, vertically oriented clay tiles (10 x 10 cm) to the north side of each mesocosm to serve as periphyton samplers.

Next, we collected and added larval amphibians to our mesocosms. From 28 to 29 May we collected 30 pairs of breeding gray treefrogs (*Hyla versicolor*) and placed them into individual containers to oviposit under a permit from the Pennsylvania Fish and Boat Commission and collection permission from the University of Pittsburgh. We then mixed the resulting eggs and moved them to 200-L wading pools containing aged well water. Once hatched, we fed the tadpoles *ad libitum* until reaching an appropriate handling size (~10 mg). On 16 June (defined as day 0 of the experiment), we added 20 gray treefrog tadpoles to each mesocosm. The densities of 10 tadpoles/species/m^2^ are well within natural densities [34]. We also set aside 20 tadpoles for staging (all tadpoles were at Gosner stage 25 [35]) and weighing (mass ± 1 SE: 11.4 ± 0.6 mg). In addition, we assessed 24-hr survival of 20 tadpoles following handling (survival was 100%). The use of amphibians and methods of preservation/euthanasia (See S1 Appendix) used in this experiment were approved by the University of Pittsburgh's IACUC.

To represent grazer communities commonly found in wetlands, we also added freshwater snails to the mesocosms. On 5 May, we collected pond snails (*Physa acuta* and *P*. *gyrina*, which can only be differentiated by dissecting their internal genitalia) and rams horn snails (*Helisoma trivolvis*) from local ponds. To prevent adult snail endoparasites from being introduced to the mesocosms, the snails used in the experiment were hatched from eggs of the snails collected from local ponds with the permits and collection permission from the Pennsylvania Fish and Boat Commission. We cultured the snails in clean well water in 200-L wading pools. On 17 June (day 1), we sorted all hatched pond snails into small (< 10 mg), medium (10 to 20 mg), and large (> 20 mg) size classes. We added five pond snails from each size class to each mesocosm. On 24 June (day 8), we sorted rams horn snails into small (< 100 mg) and large (> 100 mg) size classes (range = 17 to 211 mg) and added 4 small and 3 large rams horn snails to each mesocosm. While these snail densities are considerably lower than what can occur in wetlands in western Pennsylvania, (A.M. Turner, *unpublished data*), we added the maximum number possible to each mesocosm given the lower-than-expected number of hatchlings produced during culturing. We also assessed the 24-h survival of pond snails and rams horn snail following handling; we found 100% survival for each taxa.

### Insecticide applications

Once all animals were added, we did not disturb the mesocosms for 10 d. On 3 July (day 19), we applied the insecticide treatments using technical grade malathion (99.1% active ingredient; Chem Service Inc., West Chester, Pennsylvania, USA). Our original target concentration was 18 μg/L, which is well within the US Environmental Protection Agency’s estimated environmental concentration (EEC) for surface waters (0 to 36 μg/L; [36] and levels detected in aquatic ecosystems after the insecticide is sprayed [37]. However, 3 d after applying malathion, we assessed zooplankton abundance in 0-*Elodea* treatments and found that the insecticide reduced cladoceran abundance, but not significantly (see Results). Given that one of our goals was to determine if *Elodea* could mitigate the toxic effects of malathion on zooplankton, we decided to double the nominal concentration to 36 μg/L (which is still within the range of the EPA’s EEC values) and applied this concentration to the appropriate mesocosms on 17 July (day 37).

To achieve nominal concentrations of 36 μg/L in our tanks, we dissolved 0.88 mL of technical grade malathion (specific gravity = 1.23 g/mL) in 25 mL of ethanol to make a stock solution of (0.042 g/mL). We then added 0.71 mL of this stock solution to each appropriate mesocosm (average volume = 850 L). We elected not to apply an ethanol control because the concentrations we used have had no effect on any taxa in similar, previous experiments [12,13,38]. After applying malathion, we gently mixed the water in the mesocosms to simulate mixing that would occur during a runoff event. We also mixed control mesocosms to standardize disturbance. Whereas 36 μg/L of malathion was applied only on day 37 in the single-pulse treatment, we repeated this application procedure on days 55 and 73 for the repeated-pulse treatment. Given malathion’s rapid breakdown rate in water (t_1/2_ = 48 h at pH 8 [39]), each application in the repeated-pulse treatment represented a new exposure to our nominal malathion concentration.

We collected water samples within 1 hr of application and sent the water samples to an independent testing laboratory for concentration analysis. However, we received unreliable results from this lab and were therefore not able to verify the actual concentrations we applied. Despite these unreliable results, malathion’s effects on the cladoceran community were highly consistent with previous studies using similar nominal concentrations and application regimes [12].

### Response variables

Throughout the experiment we sampled abiotic water quality (pH, dissolved oxygen (DO), light decay rate, temperature) and biotic variables including the density of each major zooplankton group (cladocerans, copepods, rotifers), phytoplankton abundance (measured as chlorophyll *a*), and periphyton mass several times throughout the experiment. We quantified water temperature and dissolved oxygen using a calibrated digital water meter (WTW, Woburn, Massachusetts, USA) and pH using a calibrated Oakton pH 5 Acorn series sensor (Oakton Instruments, Vernon Hills, Illinois, USA). Water quality was measured by placing the meters at approximately half water depth in the center of each mesocosm. We sampled light decay rate and biotic variables using approaches described in [12] and explained in detail in S1 Appendix. Although each round of sampling took at least 3 d to complete, we hereafter identify samples by the day that sampling began (i.e. days 26, 47, 68, and 100).

To determine the influence of our treatments on periphyton grazers, we measured snail abundance and mass, as well as amphibian survival and growth. We assessed pond snail and rams horn snail abundance and average mass on day 68 by sinking five plastic cups (350 ml) with rocks in separate locations on the bottom of each mesocosm so that each cup faced upwards. We placed a single pellet of alfalfa into each cup to attract the snails. After 24 h, we removed the cups from each tank, sorted the snails by species and then counted and weighed the snails. We used this approach to sample snails because mechanically collecting snails by netting within a stovepipe sampler or on the edges of the mesocosm walls would have been confounded by the substantially higher surface area present in mesocosms with *Elodea* than mesocosms lacking *Elodea*. While the baited-trap approach that we used has its own biases (i.e. it assumes that snails in different macrophyte treatments are equally attracted to and able to access the alfalfa-baited traps), we felt that it maximized our ability to give each snail an equal opportunity of being sampled across all of our treatments. We also quantified gray treefrog survival, time to metamorphosis, and mass at metamorphosis (see S1 Appendix). The first gray treefrog metamorph emerged on day 30, just 13 d after the initial 18 μg/L malathion application (thus, no indirect effects of malathion were expected on gray treefrogs). The final gray treefrog metamorph emerged on day 94.

Finally, we used two different approaches to determine how different our macrophyte treatments were in the middle and at the end of the experiment. On day 47, we used a qualitative approach in which nine independent observers visited each mesocosm and visually ranked total *Elodea* density from 0 (no macrophytes) to 10 (most macrophytes). On day 320 (just before we took down the experiment), we quantitatively determined *Elodea* biomass density (g/m^2^) by sub-sampling macrophytes from the middle of each tank using a stovepipe sampler (radius = 0.133 m, avg. water depth = 0.381 m). We rinsed the sub-sampled macrophytes to remove any attached algae and invertebrates and then dried the macrophytes for 24 hrs at 60°C. We then weighed the plants to determine their dry mass in order to calculate *Elodea* biomass density by dividing the mass by the volume inside of the stovepipe.

### Statistical analyses

We used general linear models (GLM) to analyze the data from this experiment. To analyze the effects of *Elodea* treatment and insecticide treatment, we performed separate univariate ANOVAs on visually ranked macrophyte density at day 47 and on measured macrophyte biomass at day 320. To analyze the effects of the treatments on abiotic response variables over time, we performed a two-way repeated-measures multivariate analysis of variance (rm-MANOVA) on pH, DO, temperature and light decay. To analyze treatment effects on biotic response variables over time, we performed a two-way rm-MANOVA on cladoceran, copepod, and rotifer density, phytoplankton abundance (chlorophyll *a*), and periphyton biomass. When we found significant multivariate effects, we explored the univariate effects on each response variable using two-way rm-ANOVAs. When significant univariate time-by-treatment interactions were detected, we examined treatment effects within each time point using two-way ANOVAs. Where appropriate, we used Tukey’s test for post-hoc comparisons. This hierarchical approach allowed us to control overall experiment-wise error when performing multiple rm-ANOVAs and subsequent ANOVAs. When necessary, we log (+1) transformed our data to meet the assumptions of GLM.

To analyze the effects of the *Elodea* and insecticide treatments on snail abundance and average mass at day 68, we performed a two-way MANOVA on *Physa spp*. and *H*. *trivolvis* log + 1 abundance and average mass. We performed a separate two-way MANOVA on gray treefrog survival (arcsine-transformed), time to metamorphosis, and mass at metamorphosis. We examined all significant multivariate treatment main effects and interactions using subsequent two-way ANOVAs and Tukey’s mean comparison tests.

## Results

### *Elodea* biomass density and abiotic variables

Over the course of the experiment, *Elodea* density increased in all mesocosms containing macrophytes and the effects of our *Elodea* treatments depended on the sample date. On day 47 (i.e. ten days after the first 36 μg/L malathion application), we found no treatment effects on visually estimated *Elodea* density ranks (S2 Appendix), suggesting the *Elodea* treatments may have converged by this point. However, we discovered effects of *Elodea* treatment on final *Elodea* biomass density (day 320) and on the abiotic environment in our mesocosms. At the end of the experiment, the 10- and 50-*Elodea* treatments still did not differ in biomass density, but both contained about 50% less biomass than the 100-*Elodea* treatment. In regard to the abiotic effects, the presence of *Elodea* generally had no effect on temperature, increased DO, and maintained lower light decay rates relative to mesocosms containing no *Elodea* (see S2 Appendix for full results and figures for *Elodea* biomass density and abiotic variables). Because pH is the primary mechanism by which plants mitigate malathion’s toxicity [14], we discuss only the results for pH further here.

**Fig 1:**
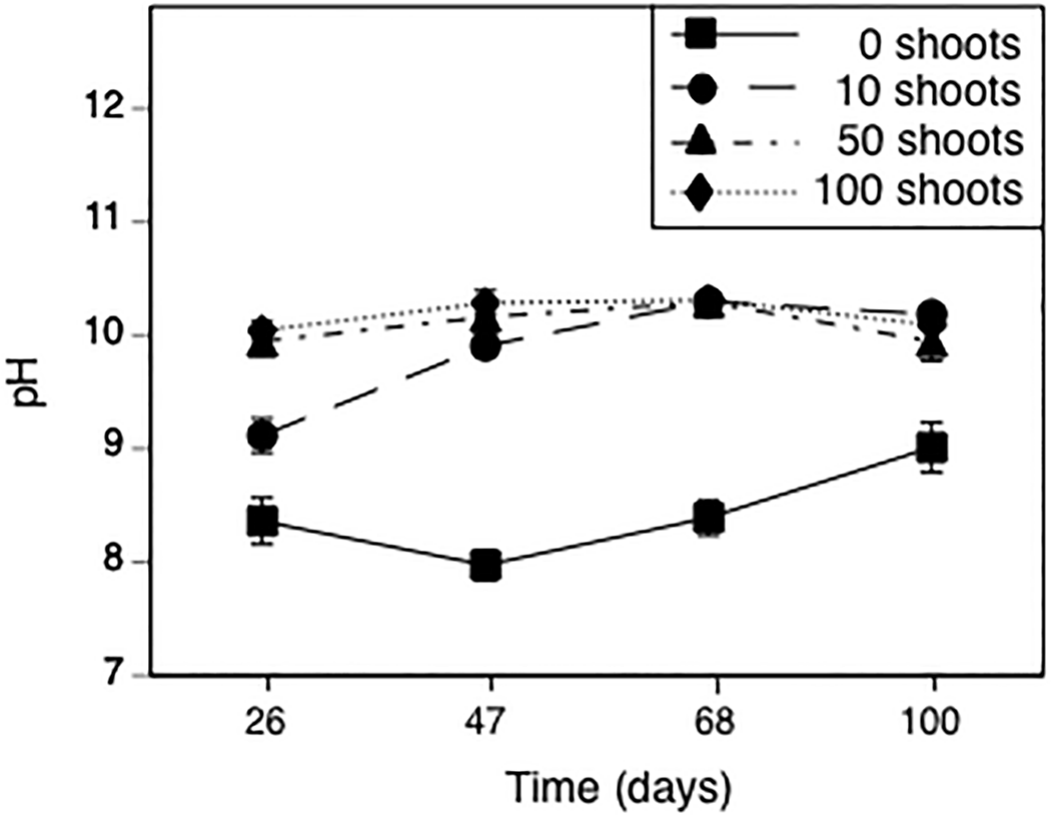
The effect of treatments planted with different initial numbers of *Elodea* shoots on pH over time. Data are means ± 1 SE.

We also observed an effect of the time-by-*Elodea* interaction on pH (*F*_9,108_ = 8.4, *p* < 0.001). At each sample date, *Elodea* treatment had an effect on pH (*F*_3,36_ = 13.5, *p* < 0.001). At day 26, pH in the 10-*Elodea* treatment was 0.76 pH units greater than the 0-*Elodea* treatment (Fig 1, *p* = 0.001), but at least 0.82 pH units less than the 50- and 100-*Elodea* treatments (all *p* < 0.001), which did not differ from each other (all *p* > 0.9). On each subsequent sample date, pH in the 10-, 50- and 100-*Elodea* treatments was at least 1.17 pH units higher than the 0-*Elodea* treatment (all *p* < 0.002) and did not differ from each other (all *p >* 0.078). Because pH is proportional to the log hydrogen ion concentration, treatments differing by 1 pH unit actually differ 10-fold in hydrogen ion concentration.

### Biotic variables

The rm-MANOVA on cladoceran, copepod and rotifer densities, phytoplankton abundance, and periphyton biomass showed significant effects of *Elodea*, insecticides, time, and all interactions (Table 1). As a result, we separately examined the time and treatment effects on each biotic response variable using two-way rm-ANOVAs (Table 2).

**Table 1:** Results of a repeated-measures MANOVA examining the multivariate effects of time, macrophyte treatment, malathion treatment, and their interactions on all biotic response variables.

A. Multivariate test (Wilks’ lambda)	df	*F*-value	*p-*value
*Elodea* (E)	15, 89	5.4	**< 0.001**
Insecticide (I)	10, 64	3.3	**0.002**
E x I	30, 130	2.1	**0.002**
Time (T)	15, 288	15.3	**< 0.001**
T x E	45, 468	2.8	**<0.001**
T x I	30, 418	1.8	**0.006**
T x E x I	90, 509	1.6	**0.001**

Bold p-values are significant at α = 0.05.

**Table 2:** Results of univariate ANOVAs examining the effects of time, macrophyte treatment, malathion treatment, and their interactions on each biotic response variables.

		Cladocerans	Copepods	Rotifers	Phytoplankton	Periphyton
B. Univariate tests	df	*P*	*p*	*p*	*P*	*p*
*Elodea* (E)	3,36	**0.002**	0.147	**< 0.001**	**< 0.001**	0.207
Insecticide (I)	2,36	**< 0.001**	0.431	0.146	0.094	0.446
E x I	6,36	**< 0.001**	0.506	0.332	**0.028**	0.860
Time (T)	3,108	**<0.001**	**< 0.001**	**< 0.001**	**0.026**	**< 0.001**
T x E	9,108	0.626	**0.008**	**< 0.001**	**0.006**	**< 0.001**
T x I	6,108	0.091	0.668	0.078	**0.005**	0.089
T x E x I	18,108	**0.001**	**0.009**	0.396	0.262	0.485

Bold p-values are significant at α = 0.05.

### Cladocerans

**Fig 2:**
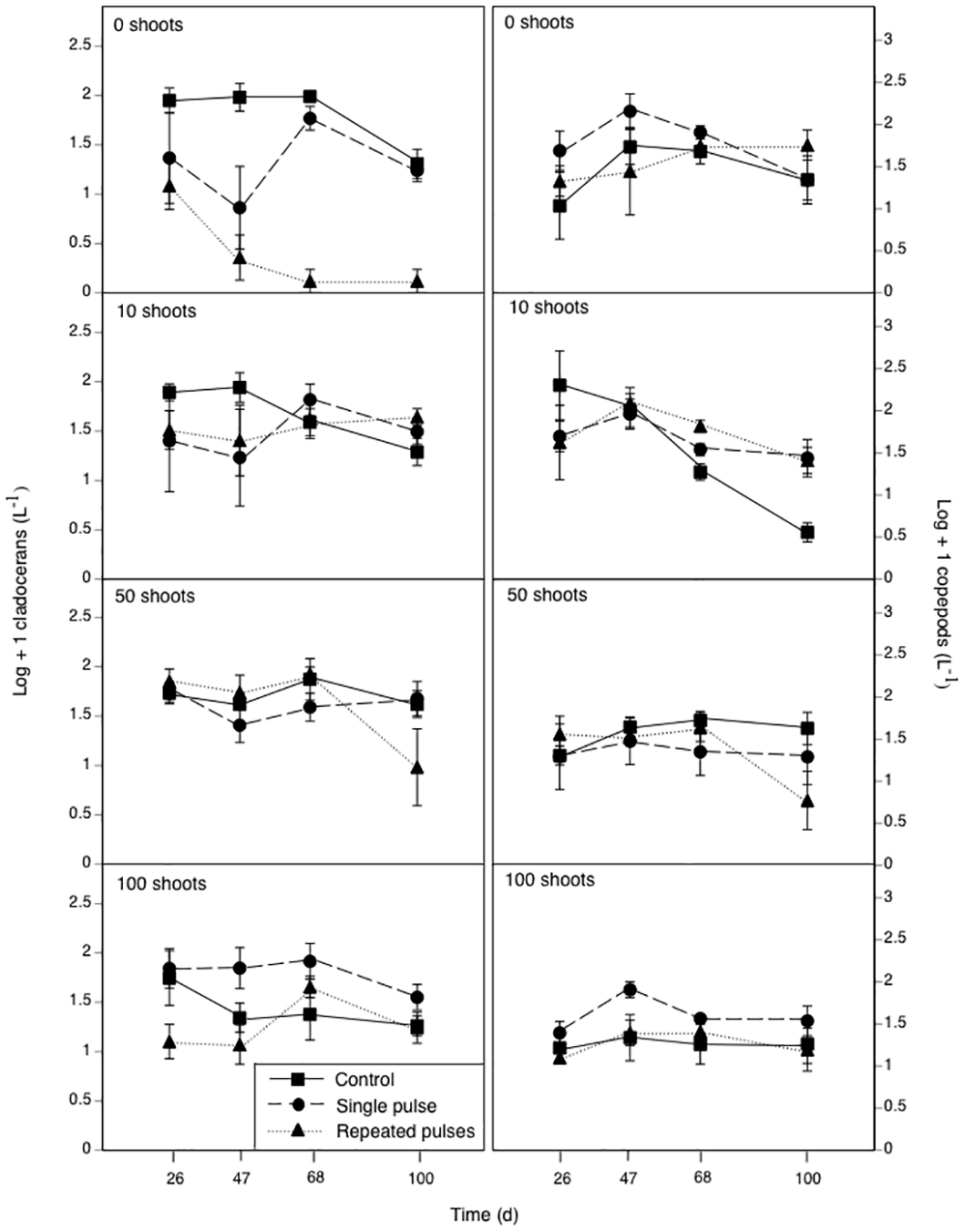
The effects of different malathion exposure scenarios in the presence of four *Elodea* treatments planted with different initial numbers of plant shoots on cladoceran density (left) and copepod density (right) over time. Data are means ± 1 SE.

Cladoceran density was influenced by *Elodea*, insecticides, the *Elodea*-by-insecticide interaction, and the three-way interaction with time (Table 2). On day 26 (i.e. after applying 18 μg/L of malathion), cladocerans were marginally affected by insecticide treatment (*F*_2,36_ = 3.0, *p* = 0.061) but not *Elodea* (*F*_3,36_ = 0.7, *p* = 0.534) or the *Elodea*-by-insecticide interaction (*F*_6,36_ = 1.2, *p* = 0.339). Because there appeared to be a pattern of different cladoceran responses to insecticide treatment within different *Elodea* treatments (Fig 2), we conducted Tukey’s mean comparisons within each *Elodea* treatment but found that single- and repeated-pulse treatments never differed from the controls (all *p* > 0.08). As noted in the methods, the lack of a malathion treatment effect in the absence of *Elodea* led to our decision to increase the malathion concentration from 18 to 36 μg/L.

On sample days 47, 68, and 100 (i.e. after malathion applications of 36 μg/L), we found that the effect of insecticide treatment on cladoceran density depended on the *Elodea* treatment (all *F*_6,36_ ≥ 3.2, *p* ≤ 0.012). In the 0-*Elodea* treatment, the single malathion exposure caused a marginally significant decline (76%) in cladocerans, relative to controls, on day 47 (*p* = 0.053). However, cladocerans returned to control levels by day 68 and remained equal to controls through day 100 (all *p* ≥ 0.302). In the repeated-pulse treatment, cladoceran densities were less than 3% of control densities on days 47, 68, and 100 (Fig 2, all *P* ≤ 0.009). However, in treatments that contained 10, 50, or 100 *Elodea* shoots, cladoceran density in the single- and repeated-pulse treatments never differed from the controls on any sample date (all *p* ≥ 0.173).

### Copepods

Copepod density was affected by time, the time-by-*Elodea* interaction and the three-way interaction with malathion (Table 2). Two-way ANOVAs revealed that on days 26 and 68, copepod density was affected by *Elodea* (*F*_3,36_ = 3.2, *p* = 0.036), but not insecticides (*F*_2,36_ = 0.2, *p* = 0.84) or their interaction (*F*_6,36_ = 1.2, *p* = 0.315). On day 26, the *Elodea* effect was driven by a 13-fold higher copepod density in the 10-*Elodea* treatment than the 100*-Elodea* treatment (Fig 2, *p* = 0.035). On day 68, however, the *Elodea* effect was driven by a 2-fold higher copepod density in the 0-*Elodea* treatment compared to the 100-*Elodea* treatment (*p* = 0.031). In between these two dates (day 47), there were no effects of *Elodea* (*F*_3,36_ = 2.7, *p* = 0.062), insecticides (*F*_2,36_ = 1.3, *p* = 0.291), or their interaction (*F*_6,36_ = 1.0, *p* = 0.446).

On day 100, we observed a *Elodea*-by-insecticide interaction (*F*_6,36_ = 2.9, *p* = 0.021) driven by an effect of insecticides on copepod density in the 10-*Elodea* treatment (*F*_2,36_ = 8.7, *p* = 0.008) but not in the other *Elodea* treatments (Fig 2, all *F*_2,36_ ≤ 2.1, *p* ≥ 0.179). With 10 *Elodea* shoots, we observed 12 to 15 times higher copepod densities in the single- and repeated-pulse insecticide treatments compared to the controls (*p* ≤ 0.016); the single- and repeated-pulse treatments did not differ from each other (*p* = 0.993).

### Rotifers

**Fig 3:**
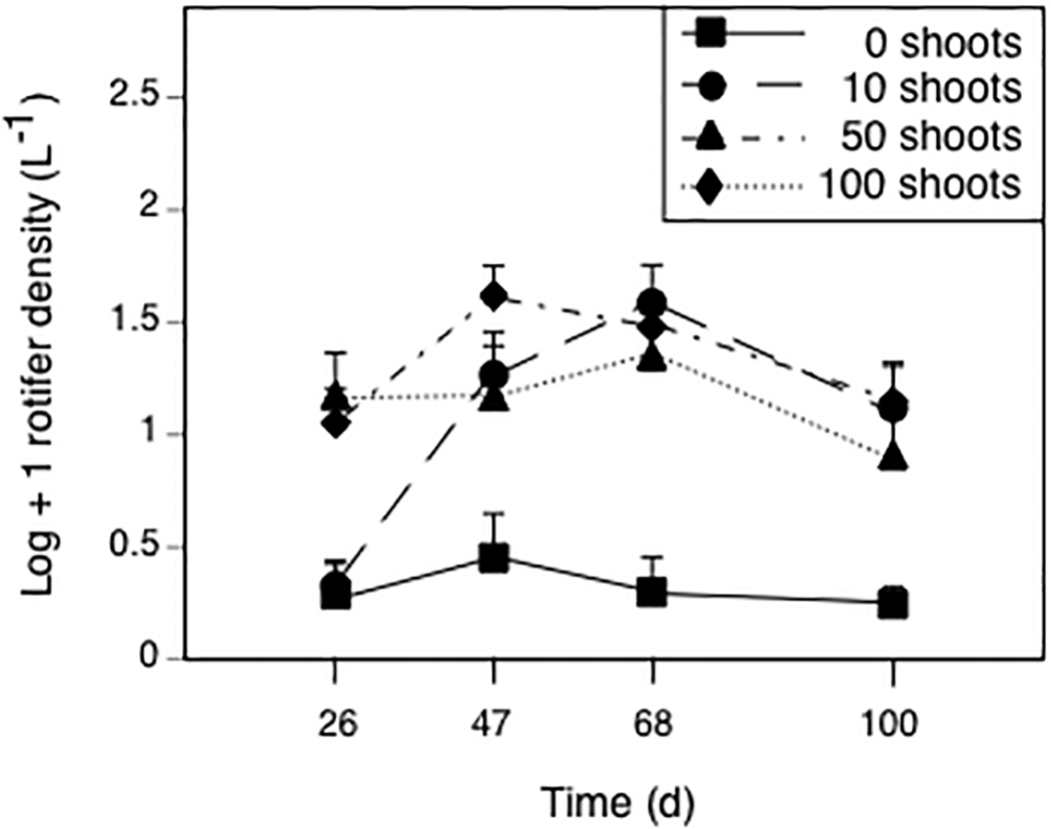
Effects of treatments planted with different initial numbers of *Elodea* shoots on rotifer density over time. Data are means + 1 SE.

Rotifer density was affected by *Elodea*, time, and the time-by-*Elodea* interaction; however, the insecticide had no effect (Table 2). Univariate effects of *Elodea* treatment on rotifer density occurred on each sample day (all *F*_3,36_ ≥ 7.1, *p* < 0.001). On day 26, the 50- and 100-*Elodea* treatments had five times higher rotifer densities than the 0-*Elodea* treatment (Fig 3, all *p* ≤ 0.004); the 10- and 0-*Elodea* treatments did not differ (*p* = 0.993). On all subsequent sampling dates, rotifer densities in the 10-, 50- and 100-*Elodea* treatments were 2 to 13 times higher than in the 0-*Elodea* treatment (all *p* ≤ 0.05).

### Phytoplankton

**Fig 4:**
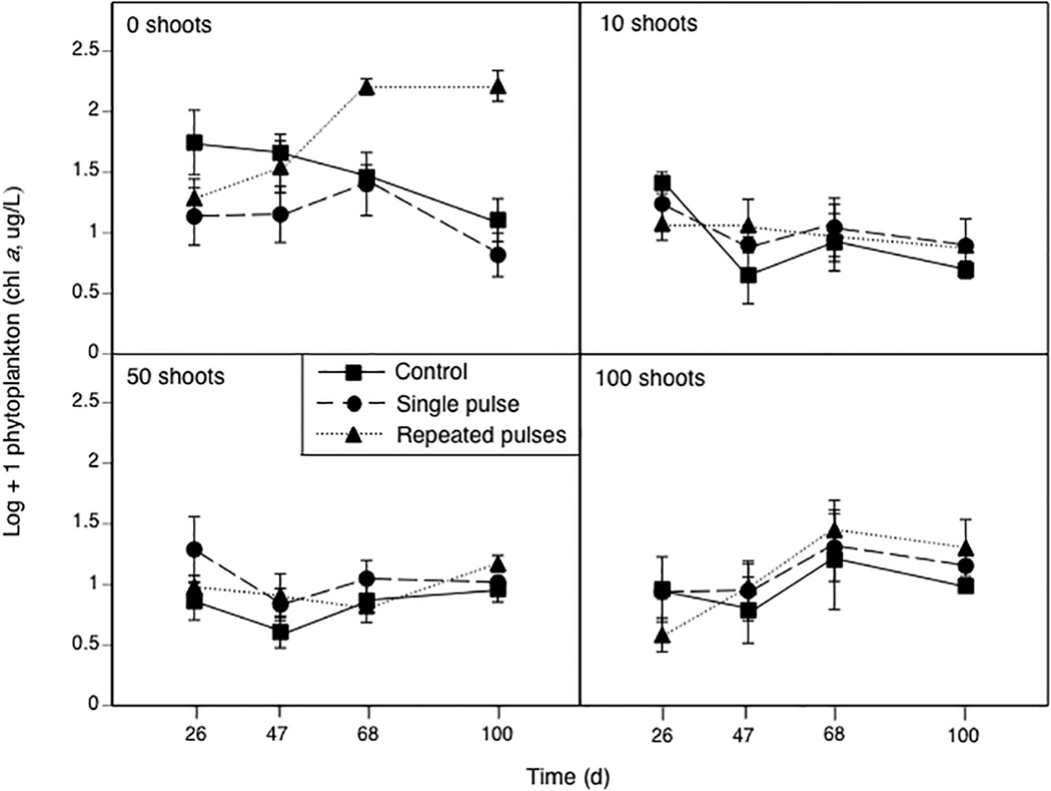
The effects of different malathion exposure scenarios in the presence of four *Elodea* treatments planted with different initial numbers of plant shoots on phytoplankton abundance (measured as chlorophyll *a*) over time. Data are means ± 1 SE.

Phytoplankton abundance was affected by *Elodea*, the *Elodea*-by-insecticide interaction, time, and several interactions with time (Table 2). As a result, we performed two-way ANOVAs on phytoplankton abundance within each sample day. On days 26, 47, and 68, we observed effects of *Elodea* treatment (all *F*_3,36_ ≥ 5.0, *p* ≤ 0.006), but not insecticides (*F*_2,36_ ≤ 2.0, *p* ≥ 0.148) or their interaction (*F*_6,36_ ≤ 1.4, *P* ≥ 0.224). On day 26, phytoplankton abundance in the 0- and 10-*Elodea* treatments was over five and two times higher, respectively, than in the 100-*Elodea* treatment (Fig 4, all *p* ≤ 0.049); abundance in the 50-*Elodea* treatment was intermediate (all *p* ≥ 0.126). On day 47, phytoplankton abundance in the 0-*Elodea* treatment was more than three times higher than the 10-, 50-, and 100-*Elodea* treatments (all *P* ≤ 0.01), which did not differ from one another (all *p* ≥ 0.874). On day 68, phytoplankton abundance in the 0-*Elodea* treatment was over five times higher than in the 10- and 50-*Elodea* treatments (all *p* ≤ 0.004); the 100-*Elodea* treatment did not differ from any of the other treatments (all *p* ≥ 0.141).

On day 100, we found an effect of insecticides on phytoplankton abundance, but the effect depended on *Elodea* treatment (*F*_6,36_ = 6.3, *p* < 0.001). This interaction occurred because insecticides had an effect on phytoplankton when *Elodea* were absent (*F*_2,36_ = 20.5, *p* < 0.001) but no effect when *Elodea* were present at any density (Fig 4, all *F*_2,36_ ≤ 2.0, *p* ≥ 0.185). In the 0-*Elodea* treatment, the insecticide effect was caused by a nearly 12-fold increase in phytoplankton abundance (i.e. a phytoplankton bloom) in the repeated-pulse insecticide treatment compared to the control and single-pulse treatments (all *p* ≤ 0.003), which did not differ from one another (*p* = 0.457).

### Periphyton

**Fig 5:**
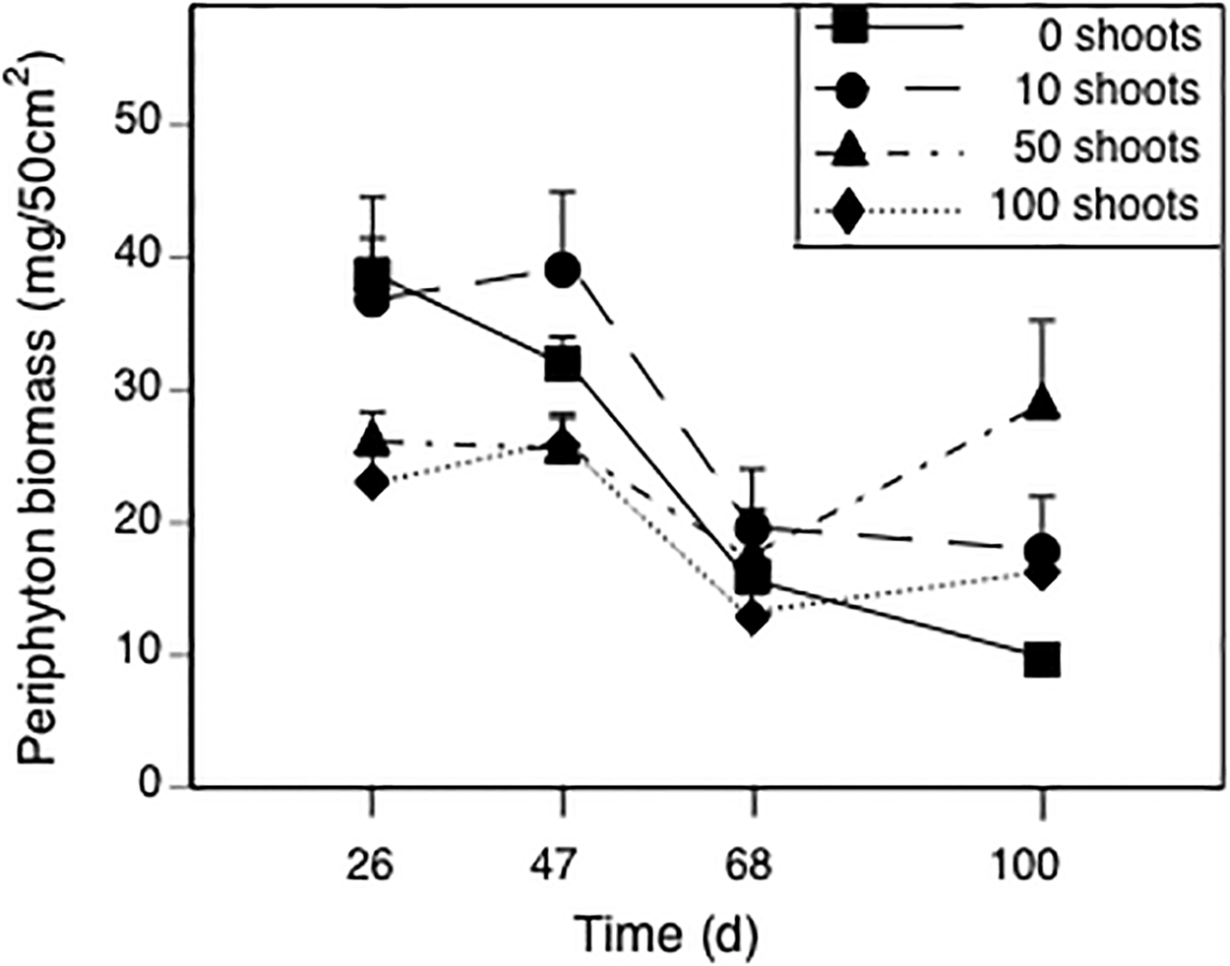
Effects of treatments planted with different initial numbers of *Elodea* shoots on periphyton biomass over time. Data are means + 1 SE.

Periphyton biomass was affected by time and the time-by-*Elodea* interaction, but not by insecticides (see Table 2). We detected effects of *Elodea* treatment on periphyton biomass on days 26, 47, and 100 (all *F*_3,36_ ≥ 3.1, *p* ≤ 0.04), but not on day 68 (*F*_3,36_ = 0.9, *p* = 0.434). On day 26, Tukey’s test revealed a trend of higher periphyton biomass in the 0- and 10-*Elodea* treatments than in the 100-*Elodea* treatment (Fig 5, all *p* ≤ 0.09), though no treatments differed from the 50-*Elodea* treatment (all *p* ≥ 0.36). On day 47, we again observed a trend of higher periphyton biomass in the 10*-Elodea* treatment than in the 50- and 100-*Elodea* treatments (all *p* ≤ 0.059), though biomass in the 0-*Elodea* treatment did not differ from any of these treatments (all *p* ≥ 0.29). Finally, on day 100, periphyton abundance in the 50*-Elodea* treatment was three times greater than in the 0-*Elodea* treatment (*p* = 0.004); the 10- and 100-*Elodea* treatments did not differ from the 0- or 50*-Elodea* treatments (all *p* ≥ 0.265).

### Snails

**Fig 6:**
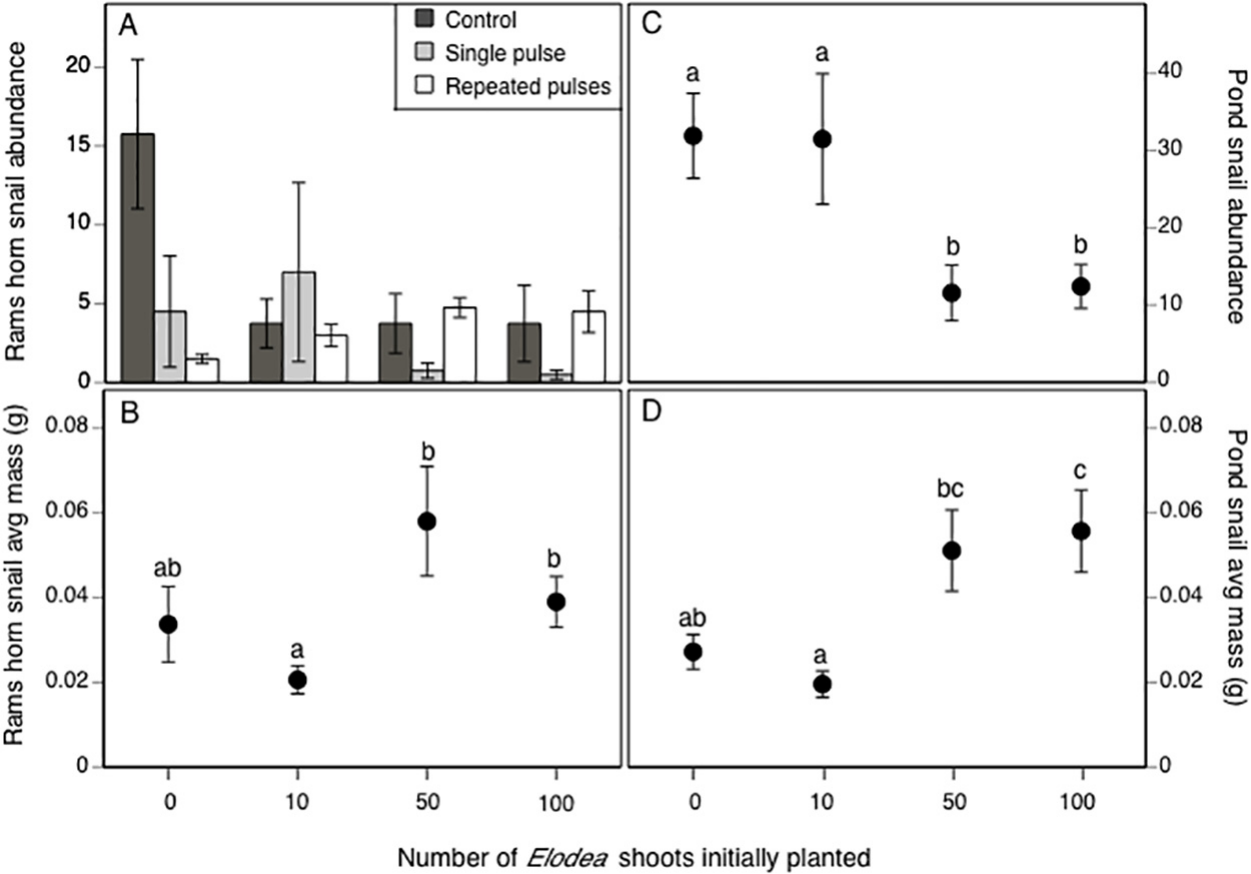
The impacts of A) the insecticide-by-*Elodea* treatment interaction on rams horn snail abundance, and *Elodea* treatment effects on B) rams horn snail average mass, C) pond snail abundance, and D) pond snail average mass. For panels B-D, different lowercase letters represent significant differences between *Elodea*-treatments (α = 0.05). Snails were sampled on day 68 using traps baited with alfalfa pellets. All data are means ± 1 SE.

The two-way MANOVA on snail abundance and mass, assessed on day 68, revealed effects of *Elodea* (Wilks’ *F*_6,70_ = 2.1, *p* = 0.026) and moderately significant effects of the *Elodea-*by-insecticide treatment interaction (Wilks’ λ, *F*_12,70_ = 1.6, *p* = 0.059), but not insecticides (Wilks’ *F*_4,70_ = 1.7, *p* = 0.111). Rams horn snail abundance was not affected by *Elodea* (*F*_3,36_ = 1.0, *p* = 0.419), but was affected by insecticides (*F*_2,36_ = 4.8, *p* = 0.014) and the *Elodea*-by-insecticide treatment interaction (*F*_6,36_ = 2.5, *p* = 0.042). In the 0-*Elodea* treatment, we observed a 10-fold decrease in abundance in the repeated-pulse treatment compared to the control, although this effect was marginally significant (Fig 6A, *p* = 0.064); abundance in the single-pulse treatment did not differ from either the repeated-pulse or control treatments (all *p* ≥ 0.116). In the 10-*Elodea* treatment, insecticides had no effect on rams horn snail abundance (all *F*_2,9_ ≤ 4.0, *p* ≥ 0.05). In the 50- and 100-*Elodea* treatments, we detected effects of insecticides (all *F*_2,36_ ≥ 4.6, *p* ≤ 0.043); rams horn snail abundance was 75% lower in single-pulse than in repeated-pulse treatments (*p* = 0.039). Controls did not differ from the single- and repeated-pulse treatments (all *p* ≥ 0.127). Rams horn snail average mass was marginally affected by *Elodea* treatment (*F*_3,36_ = 2.9, *p* = 0.052); where average mass was over 1.8 times larger in the 50- and 100-*Elodea* treatments than in the 10-*Elodea* treatment (Fig 6B, *p* ≤ 0.026), though there were no other differences between *Elodea* treatments (*p* ≥ 0.178).

Pond snail abundance was affected by *Elodea* (*F*_3,36_ = 7.5, *p* < 0.001), marginally significantly affected by insecticides (*F*_2,36_ = 3.0, *p* = 0.067), and not affected by their interaction (*F*_6,36_ = 1.7, *p* = 0.14). The *Elodea* effect was caused by a 2.5-fold higher abundance in the 0- and 10-*Elodea* treatments than in the 50- and 100-*Elodea* treatments (Fig 6C, all *p* ≤ 0.008). The insecticide effect occurred because pond snail abundance in the repeated-pulse malathion treatment was more than twice as high as in the single-pulse treatment (*p* = 0.05), though neither treatment differed from the control (all *p* ≥ 0.387). Average pond snail mass was also only affected by *Elodea* treatment (*F*_3,36_ = 6.2, *p* = 0.002) and appeared to be inversely related to pond snail abundance. Pond snails in the 50- and 100-*Elodea* treatment were > 2.6 times larger than in the 10-*Elodea* treatment (*p* ≤ 0.02). Additionally, pond snails in the 100-*Elodea* treatment were 2 times larger than the 0-*Elodea* treatment (Fig 6D, *p* = 0.031), though mass did not differ between any other *Elodea* treatments (*p* ≥ 0.110).

### Amphibians

**Fig 7:**
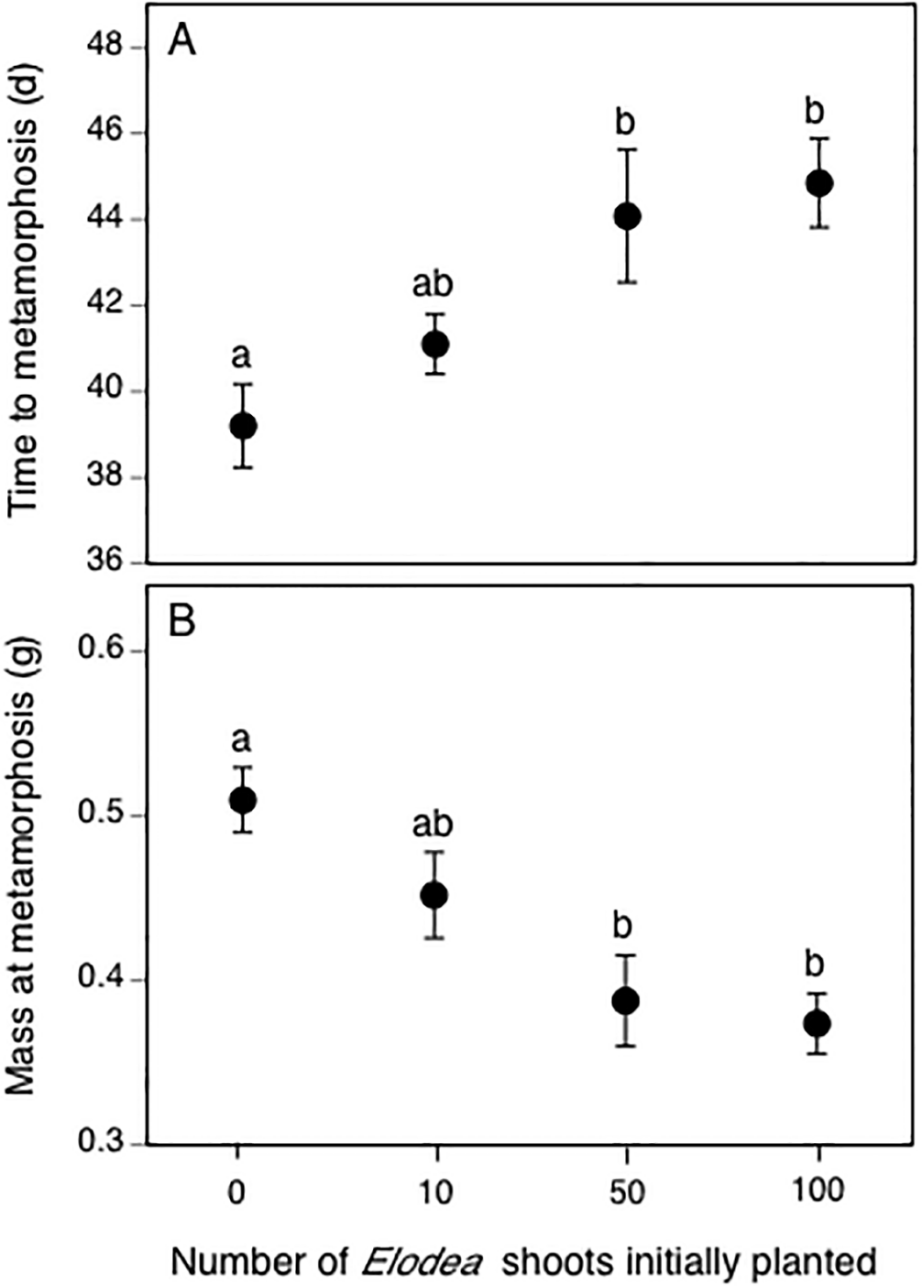
The effects of treatments planted with different initial numbers of *Elodea* shoots on gray treefrog A) time to metamorphosis and B) mass at metamorphosis. Different lowercase letters represent significant *Elodea*-treatment differences (α = 0.05). All data are means ± 1 SE.

The MANOVA on gray treefrog life history traits revealed an effect of *Elodea* (Wilks’ *F*_9,82_ = 2.29, *p* = 0.028) but no effect of insecticides (Wilks’ *F*_6,68_ = 1.0, *p* = 0.405) or their interaction (Wilk’s’ *F*_18,96_ = 0.7, *p* = 0.811). Subsequent ANOVAs revealed that survival was high across all treatments (mean ± 1 SE; 86 ± 2%) and unaffected by *Elodea* (*F*_3,36_ = 0.6, *p* = 0.629). However, *Elodea* treatment affected mass at metamorphosis (*F*_3,36_ = 6.6, *p* = 0.001) and time to metamorphosis (*F*_3,36_ = 5.6, *p* = 0.003). Compared to the 0-*Elodea* treatment, time to metamorphosis did not differ in the 10-*Elodea* treatment (*p* = 0.621) but took 5 d longer in the 50- and 100-*Elodea* treatments (Fig 7A, all *p* ≤ 0.02). For mass at metamorphosis, gray treefrog raised with 0-*Elodea* were similar in mass to those raised with 10 *Elodea* shoots (all *p* > 0.348), but mass in the 50- and 100-*Elodea* treatments was approximately 25% lower (Fig 7B, all *p* ≤ 0.007).

## Discussion

We tested the general hypothesis that the submerged macrophyte *Elodea canadensis* would mitigate the direct and indirect effects of several realistic insecticide exposure scenarios in aquatic communities. Overall, we found that whenever *Elodea* was present, malathion’s direct effects were strongly mitigated. This mitigating effect occurred regardless of whether insecticide exposure occurred as single or repeated pulses. By buffering cladocerans from malathion’s direct lethal effects, *Elodea* also dampened the insecticide’s cascading effects on the rest of the community. Further, we discovered that 50- and 100-*Elodea* treatments suppressed the biomass of periphyton, resulting in reduced snail abundance and tadpole growth compared to treatments initially planted with 0 or 10 *Elodea* shoots.

An important prediction in our experiment was that malathion would decimate sensitive cladocerans in the absence of *Elodea* but this effect would be mitigated in the presence of *Elodea* in a density-dependent manner. Indeed, we discovered that without *Elodea*, both single and repeated malathion applications of 36 μg/L reduced cladoceran densities relative to insecticide-free controls. Although cladocerans recovered to control levels within 3 wks after the single pulse exposure, the repeated pulse exposures maintained low cladoceran densities for the duration of the experiment. This result is consistent with reported cladoceran sensitivities to malathion in laboratory experiments (lethal concentration required to kill 50% of animals; LC50_48h_ < 5 μg/L [40], PAN Pesticide Database, http://www.pesticideinfo.org). Other studies conducted in mesocosms have demonstrated similarly toxic effects of comparable malathion concentrations on cladoceran populations [12,13].

In contrast to malathion’s high toxicity in the absence of *Elodea*, the single- and repeated-pulse malathion treatments had no effect on cladocerans in any *Elodea* treatment containing plants. Using microcosm experiments, Brogan & Relyea [30] found that a similar range of realistic *E*. *canadensis* biomass densities to those achieved in our study (i.e. range = 177 to 747 g dry weight/m^3^; [41]) reduced malathion’s toxicity to *Daphnia magna* in a density-dependent manner, with the highest *Elodea* densities increasing the insecticide’s LC50_48-h_ value by six times. These effects appear to be generalizable across most macrophyte species [31] because mitigation is primarily driven by the elevated water pH caused by plant photosynthesis [14]. While wetlands often range from pH 5 to 8 [42], pH levels of 9 and above are not uncommon in dense macrophyte beds [43,44], particularly in the canopy near the surface [45,46]. It should also be noted that high pH levels can also occur during algal blooms (phytoplankton and/or periphtyon; e.g., [47]). Thus, malathion’s toxic effects would also likely be reduced under these conditions. Of course, other aspects of water chemistry (alkalinity, water hardness, clarity, etc.) can strongly influence pH value and variability in natural water bodies [15] and need to be considered when predicting pesticide effects. Nevertheless, our discovery that realistic macrophyte densities mitigate insecticide toxicity under the more realistic conditions in the present study suggests that this ability may translate to the field, though testing this conclusion is an important next step.

Compared with cladocerans, rotifers are highly resistant to malathion [40]. Previous mesocosm studies have also shown that compared to cladocerans, copepods are relatively resistant to malathion concentrations similar to those applied in our experiment [12,13,48]. Thus, the lack of any direct effects of malathion on copepods and rotifers in our study was not surprising. In fact, cladoceran declines following malathion exposure in the absence of *Elodea* often result in the increased abundance of copepods and rotifers due to competitive release [12,13,49]. However, we found no evidence of this indirect effect in our study. In fact, the only case where we observed significantly higher copepod densities following malathion applications (in both the single- and repeated-pulse treatments) was on day 100 in the 10-*Elodea* treatment, where malathion treatment had no effect on cladoceran density at any earlier sample dates.

Although malathion had only minor effects on copepods and rotifers, we observed effects of *Elodea* treatment on these taxa. Copepods were generally less abundant in the 100-*Elodea* treatment than in the 0- and 10-*Elodea* treatments throughout the experiment, though this relationship depended on the sample date. It is possible that copepod density in the 10- and 50-*Elodea* treatments differed at certain time points (e.g., day 100) due to lag effects of early differences in *Elodea* treatments (i.e. before day 47), including differences in phytoplankton species composition. However, we did not design our experiment to test such species-level interactions. To our knowledge, no studies have examined mechanisms by which submerged macrophytes might suppress copepod populations. In contrast, rotifers were generally more abundant whenever macrophytes were present. This is likely the result of macrophytes providing rotifers with an important refuge from predators, such as cyclopoid copepods [50].

We also predicted that cladoceran declines following malathion exposure would initiate phytoplankton blooms. When we examined phytoplankton abundance in the malathion single-pulse treatment, we found no effects of *Elodea* treatment, likely because the insecticide caused only ephemeral (< 3 wk) cladoceran declines. In the repeated-pulse treatment, however, consistently low cladoceran densities occurred when *Elodea* was absent and these caused phytoplankton blooms that developed by day 68 and persisted through day 100. However, these blooms did not occur whenever the macrophyte was present due to the mitigating effects of *Elodea* on cladocerans. Thus, we found support for our hypothesis that *Elodea* would not only mitigate the direct effects of malathion on cladocerans, the macrophyte would also mitigate the subsequent indirect effects on phytoplankton.

The cascading effects that insecticides have in *Elodea*-free aquatic communities are becoming well established. For example, Relyea & Diecks [12] documented phytoplankton blooms in outdoor mesocosms following repeated, but not single, applications of low malathion concentrations (10 μg/L) because cladocerans were kept at low abundance for several weeks. Other studies have documented phytoplankton blooms following repeated-pulse or press insecticide applications, but only where concentrations were high enough to apparently cause local extinctions of cladoceran populations [12,28,51]. While phytoplankton blooms are observed following exposure to many different insecticides, the primary mechanism is typically due to dramatic declines in the abundance of cladocerans due to direct insecticide toxicity [4]. Thus, the present study, which investigated the ecological factors capable of partially or completely mitigating such cascades, has clear conservation and management implications for developing better strategies to protect contaminated freshwater ecosystems.

Despite the sustained phytoplankton blooms in the 0-*Elodea*, repeated-pulse malathion treatment in this study, we did not find support for our prediction that the phytoplankton blooms would reduce periphyton mass via competition for light and nutrients. Instead, we found no effects of malathion on periphyton, regardless of *Elodea* treatment. However, we would expect this result if, across malathion treatments, grazing pressure was consistently above a threshold level necessary to prevent periphyton mass from increasing beyond a minimum mass. Under such conditions, one would expect that, instead of creating differences in periphyton mass, the phytoplankton blooms would actually manifest as differences in the abundance of grazers in different malathion treatments, possibly driven by changes in periphyton quality or production [52].

In contrast to periphyton, the trophic cascade initiated by malathion in the absence of *Elodea* appeared to negatively affect rams horn snail abundance. Without *Elodea*, the repeated-pulse malathion treatment tended to decrease rams horn snail abundance relative to controls. Given that rams horn snails (and gastropods in general) exhibit low sensitivity to malathion (LC50_48h_ = 500,000 μg/L [53]) it is unlikely that the insecticide had any direct effects on the snails. Instead, the adverse effects of malathion-induced phytoplankton blooms may have decreased periphyton productivity or quality and ultimately manifested as reduced snail abundance. However, it is important to note that rams horn snails were the only grazer affected by malathion in the absence of *Elodea* and the reasons for this are unclear.

While a major focus of the present study was on the influence of *Elodea* on malathion’s community-level effects, we also discovered important and novel effects of the macrophyte on community structure. For example, during the first two sampling dates (days 26 and 47), periphyton biomass was generally higher in tanks with 0 or 10 macrophytes than 50 or 100 macrophytes. This pattern makes sense as macrophytes and periphyton overlap spatially and compete for light and nutrients in the benthos [54]. Because periphyton is a primary food source for many grazer species, we predicted that such competitive interactions would have important implications for the growth and abundance of tadpoles and snails [18,55].

Indeed, we found that *Elodea* had pronounced effects on grazer community structure. For example, pond snail abundance was closely related to periphyton biomass early in the experiment, with the highest abundances occurring in the 0- and 10-*Elodea* treatments and lower abundances occurring in the 50- and 100-*Elodea* treatments. While this response may be related to food availability or some other ecological interaction, it is also possible that the snail’s chemosensory ability to detect the alfalfa pellets used in our passive sampling traps was inhibited by the elevated pH in the 50- and 100-*Elodea* treatments [56]. Consistent with *Elodea’s* effects on snail abundance, we also observed that pond snails and rams horn snails generally had larger average masses in the higher *Elodea* treatments (50- and 100-*Elodea* shoots initially planted) than in the lower *Elodea* treatments (0- and 10-*Elodea* shoots initially planted). The trend of greater average snail mass at higher *Elodea* densities is likely the result of the lower numbers of grazers in these treatments and, thus, higher *per capita* resource availability. Although it is possible that snails grew to larger sizes in the higher *Elodea* treatments because they were eating the plants, freshwater snails primarily graze algae and are not considered to be important herbivores on living macrophyte tissues [57,58].

Higher *Elodea* treatments also had adverse effects on amphibians, causing gray treefrogs to emerge later and at a smaller mass. As in the case of pond snails, this is likely a result of increased competition for resources driven by the negative effects of higher *Elodea* biomass densities on periphyton biomass. An additional possibility is that periphyton quality decreased as *Elodea* biomass density increased, but the few experiments addressing this question have found no effect of macrophytes on periphyton quality [59,60]. Regardless of the mechanism, the reduced growth and prolonged larval developmental period experienced by gray treefrogs has important implications because anurans that metamorphose later and at smaller masses experience reduced survival to reproduction and recruitment [61,62]. More studies examining how different habitats (e.g., macrophyte-free versus macrophyte-dominated) and exposure to anthropogenic contaminants might interact to influence the survival and life-history traits of amphibians are needed as these taxa continue to decline worldwide [63–65].

## Conclusions

The present study demonstrates the clear ability of submerged macrophytes to mitigate the toxicity of the widely used insecticide malathion in complex communities and under various realistic exposure scenarios. While the mitigating influence of submerged macrophytes on malathion’s toxicity is now well established, several important questions remain. For example, one important next step is to test whether our results scale up to field, where more complex ecological interactions and environmental conditions are present. Further, the relative strength of submerged plant effects on the toxicity of different commonly applied insecticides needs to be examined to determine if there are particular insecticide properties that lead to differential mitigation success. The results of such studies could help improve best management practices to mitigate surface runoff containing pesticides. Finally, given our finding that macrophytes suppressed the growth and abundance of several benthic species, more studies are needed to examine the costs and benefits of different submerged macrophyte densities on aquatic communities, both in the presence and absence of contaminants such as pesticides. This will help aquatic resources managers determine ideal macrophyte densities for maximizing biodiversity and ecosystem function in potentially contaminated environments.

## Supporting Information

S1 Appendix Additional sampling details for response variables. (DOCX)

S2 Appendix Additional response variable results. (DOCX)

S3 Appendix Zooplankton species list. (DOCX)
