# Supplementary material for: Submerged Macrophytes Mitigate Direct and Indirect Insecticide Effects in Freshwater Communities
Source: PLoS One. 2015 May 15;10(5):e0126677. doi: 10.1371/journal.pone.0126677 (PMC4433326; doi:10.1371/journal.pone.0126677)
Supplement: S1 Appendix — (DOCX) [file pone.0126677.s001.docx]

**S1 Appendix. Additional sampling details for response variables**

*Sampling abiotic variables*

We quantified the shading effect of phytoplankton on periphyton by measuring the rate of light decay with depth. Using an underwater quantum sensor (LI-COR, Lincoln, Nebraska, USA), we measured photosynthetically active radiation at 10 and 20 cm below the water surface by positioning the light meter so no macrophyte shoots were shading the sensor. To calculate light decay rate (*K*), we used the formula:

$$K=\frac{\ln(L_{10}/L_{20})}{d}$$

where *L*_10_ equals the light intensity at 10 cm, *L*_20_ equals the light intensity at 20 cm, and d is the difference in depth between those two measurements.

*Sampling zooplankton*

Using methods from Relyea & Diecks (2008), we collected zooplankton and identified them to species. We collected zooplankton samples using a 0.2-L tube sampler that was plunged approximately 0.25 m deep at five locations in each mesocosm and the water was filtered through a 62-µm Nitex screen. Zooplankton samples were preserved in 30% ethanol for enumeration and identification to species. We identified a total of 18 zooplankton species in the experiment but we ultimately grouped them as cladocerans, copepods, and rotifers because species within each group exhibited very similar responses to our treatments; similar results have been found in past experiments (Relyea and Diecks 2008, Relyea and Hoverman 2008, Hua and Relyea 2012).

*Sampling phytoplankton*

We also quantified phytoplankton following the protocols of Relyea & Diecks (2008). To sample phytoplankton, we plunged plastic cups approximately 5 cm under the water surface to collect 500 ml of water from each mesocosm. The water was vacuum-filtered through a Fisherbrand GF/C G4 filter (4.25 cm diameter, particle retention = 1.2 µm). Samples were wrapped in foil and frozen until chlorophyll *a* analysis. We analyzed all chlorophyll *a* samples within 30 d of collection using methods modified from Arar and Collins (1997)*.* We used a mortar and pestle to grind the filters in 90% acetone and steeped the samples in the dark for 24 hrs at 3˚C. We then centrifuged the samples for 30 sec at 12,000 rpm and determined the concentration of chlorophyll *a* using a flurometer (TD-700, Turner Designs Inc., Sunnyvale, California, USA).

*Sampling periphyton*

We measured periphyton by removing a single vertically oriented clay tile from each mesocosm. We scrubbed and rinsed the periphyton from a standardized area (10 x 5 cm) of each clay tile (i.e. the half of each tile that was closest to the water surface). We then collected and vacuum-filtered the algae water onto a pre-weighed Fisherbrand GF/C filter (7.0 cm diameter, particle retention = 1.2 µm) that had been dried for 24 h at 60˚C. After filtration, we dried the filters for another 24 hrs and re-weighed them to determine periphyton biomass.

*Gray treefrog metamorph collection and processing*

We collected gray treefrogs as they metamorphosed to compare survival and larval development. After the first metamorph emerged (day 30), we checked mesocosms daily for metamorphs. Once collected, we held the metamorphs in the lab in separate containers (one container/mesocosm) until tail resorption (Gosner stage 46; Gosner 1960). Once tail resorption was complete, we euthanized the metamorphs in 2% MS-222 (tricaine methane sulfonate) and preserved them in glass jars containing 10% buffered formalin, allowing us to subsequently assess metamoprh mass at metamorphosis in addition to survival and time to metamorphosis.

**References (contained in S1 Appendix only)**

Arar EJ, Collins GB. *In vitro* determination of chlorophyll *a* and pheophytin *a* in marine and freshwater algae by fluorescence. Method 445.0. National Exposure Research Laboratory, USEPA, Cincinnati, Ohio, USA; 1997.

Hua, J, Relyea RA. East Coast versus West Coast: Effects of an insecticide in communities containing different amphibian assemblages. Freshw Sci 2012;21: 787-799.
