# Supplementary material for: Submerged Macrophytes Mitigate Direct and Indirect Insecticide Effects in Freshwater Communities
Source: PLoS One. 2015 May 15;10(5):e0126677. doi: 10.1371/journal.pone.0126677 (PMC4433326; doi:10.1371/journal.pone.0126677)
Supplement: S2 Appendix — (DOCX) [file pone.0126677.s002.docx]

**S2 Appendix. Results for macrophyte biomass and abiotic variables.**

*Treatment effects on macrophyte biomass*

Over the course of the experiment, *E. canadensis* density increased in all mesocosms containing macrophytes. The univariate ANOVA on the visually estimated biomass ranks collected on day 47 revealed no effects of macrophyte density (*F*_2,27_ = 2.4, *P =* 0.108), insecticides (*F*_2,27_ = 1.3, *P =* 0.295), or the macrophyte-by-insecticide interaction (*F*_2,27_ = 0.2, *P =* 0.922). When we quantified macrophyte biomass on the last day of the experiment (i.e. day 320), we detected significant effects of macrophyte treatment (*F*_2,27_ = 5.7, *p =* 0.008) and insecticides (*F*_2,27_ = 3.7, *p =* 0.039), but not the interaction (*F*_4,27_ = 0.3, *p =* 0.886). Tukey’s tests revealed that the macrophyte treatment effect was driven by an approximately 50% greater *E. canadensis* biomass in the 100-macrophyte treatment compared to the 10- and 50-macrophyte treatments (Fig. S2.1A, all *p ≤* 0.02); the latter two treatments did not differ from each other (*P* = 0.998). The insecticide effect was caused by an approximately 50% greater *E. canadensis* biomass in the repeated-pulse treatment than in the control (*p =* 0.03); the single-pulse treatment did not differ from the control or repeated-pulse treatments (Fig. S2.1B; all *p* ≥ 0.339).

This increase in *E. canadensis* density in repeated-pulse treatments could be a result of the repeated inputs of phosphorus provided by each addition of the organophosphate insecticide, malathion. However, the ability of microorganisms to remineralize nutrients contained in insecticide molecules has received little attention to draw definitive conclusions (but see Omar 1998). A second possibility is that with each malathion application, a new source of nutrients was available in the form of dead cladocerans, where the decomposition of the carcasses could recycle nutrients and facilitate macrophyte growth. However, our study was not designed to elucidate the mechanism driving this pattern.

*Treatment effects on abiotic variables*

The rm-MANOVA on temperature, pH, dissolved oxygen, and light decay revealed multivariate effects of macrophyte treatment, the macrophyte-by-insecticide interaction, time, and the time-by-macrophyte interaction (Table S2). Because of the significant multivariate time-by-macrophyte interaction, we examined the univariate time-by-macrophyte interaction effects on each response variable (pH results discussed in main text). Where appropriate, we subsequently examined the univariate macrophyte treatment effects within each sample date.

Average daytime water temperatures were (mean ± 1 SE) 20.8 ± 0.07 °C, 20.6 ± 0.06 °C, 22.6 ± 0.08 °C, and 19.6 ± 0.08 °C on days 26, 47, 68, and 100, respectively. However, we did not observe a time-by-macrophyte interaction (*F*_9,108_ = 0.3, *p* = 0.99) or a macrophyte-by-insecticide interaction (*F*_6,36_ = 1.1, *p* = 0.38) on water temperature.

Dissolved oxygen was significantly influenced by the time-by-macrophyte interaction (*F*_9,108_ = 2.7, *p* = 0.009). We found significant macrophyte treatment effects on dissolved oxygen concentrations at each sample date (all *F*_3,36_ > 9.1, *p <* 0.001). Tukey’s mean comparisons tests revealed that on all sample dates, dissolved oxygen did not differ among the 10-, 50- and 100-macrophyte treatments (all *p ≥* 0.4), but was at least 30% greater in these treatments than in the 0-macrophyte treatment (Fig. S2.2A, all *p* ≤ 0.002;).

Light decay rate was also influenced by the time-by-macrophyte interaction (*F*_9,108_ = 4.2, *p* < 0.001). While there was no effect of macrophyte treatment on light decay on day 26 (Fig. S2.2B, *F*_3,48_ = 0.4, *p* = 0.751), each subsequent sample date revealed a significant macrophyte effect (all *F*_3,48_ > 4.9, *p* < 0.006). Tukey’s mean comparisons test revealed that at day 47, the light decay rate in the no-macrophyte treatment was 70% higher than in the 100-macrophyte treatment (*p* = 0.006), but the 10- and 50-macrophyte treatments did not significantly differ from the 0- or 100-macrophyte treatments (all *p* ≥ 0.07). On days 68 and 100, light decay rate in the 0-macrophyte treatment was at least 44% greater than in the 10-, 50-, and 100-macrophyte treatments (all *p* < 0.001), which did not differ from each other (all *p* ≥ 0.73).

**References (contained in S2 Appendix only)**

Omar SA. Availability of phosphorus and sulfur of insecticide origin by fungi. Biodegradatio*n* 1998;9: 327-336.

**Tables**

Table S2. Results of repeated measures MANOVA on water temperature, pH, dissolved oxygen and light decay in mesocosms treated with a factorial combination of four macrophyte densities and three insecticide (malathion) application regimes. Bold p-values are significant at p < 0.05.

| **Source (Wilk's lambda)** | **df** | ***F*-value** | ***p-*value** |
| --- | --- | --- | --- |
| Macrophyte | 12, 88 | 14.5 | **< 0.001** |
| Insecticide | 8, 66 | 1.3 | 0.265 |
| Macrophyte x insecticide | 24, 116 | 1.7 | **0.037** |
| Time | 12, 278 | 54.3 | **< 0.001** |
| Time x macrophyte | 36, 395 | 3.1 | **0.001** |
| Time x insecticide | 24, 368 | 1.5 | 0.057 |
| Time x macrophyte x insecticide | 72, 415 | 1.3 | 0.06 |

**Figure legends**

Figure S2.1. The effect of A) number of macrophyte shoots planted and B) insecticide treatment on final *E. canadensis* biomass as measured on day 320. Different lower case letters show significant differences (α = 0.05). Data are means ± 1 SE and exclude treatments containing no macrophytes.

Figure S2.2. The effect of macrophyte density on (A) dissolved oxygen and (B) light decay over time (means ± SE).
