# Supplementary material for: Submerged Macrophytes Mitigate Direct and Indirect Insecticide Effects in Freshwater Communities
Source: PLoS One. 2015 May 15;10(5):e0126677. doi: 10.1371/journal.pone.0126677 (PMC4433326; doi:10.1371/journal.pone.0126677)
Supplement: S3 Appendix — (DOCX) [file pone.0126677.s003.docx]

**S3 Appendix. List of zooplankton species sampled in mesocosms.**

Table S3. Relative abundance of zooplankton species collected from mesocosms across all four sampling dates (days 26, 47, 68, and 100).

| Group | Species name | % Abundance |
| --- | --- | --- |
| **Cladocerans** | *Chydorus sphaericus* | 20.8 |
|  | *Daphnia pulex* | 12.4 |
|  | *Scapholeberis mucronata* | 2 |
|  | *Daphnia ambigua* | 0.5 |
|  | *Simocephalus vetulus* | 0.2 |
|  | *Bosmina longirostris* | 0.2 |
| **Copepods** | *Cyclopoid nauplii spp.* | 24.9 |
|  | *Skistodiaptomus oregonensis* | 8.2 |
|  | *Microcyclops rubellus* | 7.6 |
|  | *Calanoid nauplii spp.* | 3.3 |
| **Rotifers** | *Monostyla quadridentata* | 13.6 |
|  | *Notholca foliaceae* | 1.9 |
|  | *Platyius palutus* | 1.6 |
|  | *Lecane mira* | 1.5 |
|  | *Euchlanis dilatata* | 1 |
|  | *Monostyla closterocerca* | 0.2 |
|  | *Keratella earlinae* | 0.1 |
